# A possible rising incidence of malignant germ cell tumours in young women.

**DOI:** 10.1038/bjc.1984.104

**Published:** 1984-05

**Authors:** A. H. Walker, R. K. Ross, M. C. Pike, B. E. Henderson


*Br. J. of Cancer* (1984), **49**, 669–672

### Short Communication

### A possible rising incidence of malignant germ cell tumours in young women

A.H. Walker, R.K. Ross, M.C. Pike & B.E. Henderson

*Department of Preventive Medicine, University of Southern California, School of Medicine, 2025 Zonal Avenue, Los Angeles, California 90033 USA.*

Germ cell tumours comprise 95 percent of malignant testicular tumours but less than two percent of malignant ovarian tumours (Teilum, 1976; Weiss *et al.*, 1977). Further, the incidence rate of malignant testicular tumours is nearly 10 times higher than the rate for malignant ovarian germ cell tumours and it is one of the most common cancers in young men. As a result, the epidemiology of testicular germ cell tumours has been more extensively studied and more is known about associated risk factors than for the comparable tumours in females. An increasing incidence of testicular tumours in whites during this century has been well documented, both in the U.S. (Ross *et al.*, 1979; Schottenfeld, *et al.*, 1980) and elsewhere (Clemmeson, 1968; Davies, 1981). The recent development of population based tumour registries reporting data by histologic type now permits an assessment of short term secular trends, as well as other epidemiologic features, of malignant ovarian germ cell tumours. Los Angeles County data indicate that the increasing incidence of testicular germ cell tumours may be paralleled in females.

The Los Angeles County/University of Southern California Cancer Surveillance Program (CSP) is a population based tumour registry that attempts to identify all newly diagnosed cancer cases among the seven million residents of the County. A detailed description of the methodology, organization, and administration of the CSP has been given elsewhere (Hisserich *et al.*, 1975; Mack, 1977). Populations at risk by age, race and sex are based on U.S. Census data. The CSP makes adjustments to the Los Angeles County census data to account for postcensal change and under counting. A socio-economic status (SES) index, ranging from a low of 5 to a high of 1, is assigned to each census tract of the County, using a modification of the two-factor Hollingshead Index (Henderson *et al.*, 1975). CSP case ascertainment is currently complete for the 10 year period 1972–1981, during which time over 95 percent of Los Angeles County incident cases have been registered.

Due to the small number of cases of malignant ovarian germ cell tumours occurring annually, we dichotomized this 10 year period into two 5-year periods in order to examine trends in incidence for two major histologic categories of germ cell tumours (Table I). The two major categories are: (1) the germinomas, including seminoma of the testis and dysgerminoma of the ovary, and (2) the teratomas, including both embryonal and extra-embryonal cell types (i.e. embryonal cell carcinoma, endodermal sinus tumour, and choriocarcinoma) (Novak and Woodruff, 1979). Sizeable increases in the incidence of malignant germ cell tumours of both histologic types occurred in the 15–34 age range in both sexes between the two 5-year calendar periods (1972–76 and 1977–81). These trends are statistically significant in the 25–34 age group for both males and females. One difference between the sexes was that the largest increases occurred in the germinoma group for females, while, for males, the largest and most highly significant increases were in the teratoma group.

We compared the secular trends for the ovarian tumours (of both histologic types combined) in Los Angeles with data obtained from the Surveillance, Epidemiology, and End Results (SEER) Program, a series of population based tumour registries covering 10 percent of the total U.S. population (Young, 1983 personal communication). SEER data from 1973–1980 (dichotomized into the periods 1973–76 and 1977–80) also show substantial increases in incidence rates for the same age categories (15–24 and 25–34 years of age,) although the magnitude of the increase is not as large as in Los Angeles. Specifically, from the SEER data, incidence rates (per 1,000,000) increase from 7.2 ($N=53$) to 8.6 ($N=68$) in the 15–24 age group and from 3.3 ($N=19$) to 5.7 ($N=39$) in the 25–34 age group.

Correspondence: A.H. Walker

Received 3 February 1984; accepted 28 February 1984.

© The Macmillan Press Ltd., 1984

**Table I** Average annual age-specific incidence rates of malignant testicular and ovarian germ cell tumours by histologic type: Los Angeles County, 1972–76 and 1977–81

| Site of germ Cell tumour and age group | *Germinomas* 1972–76 Rate | (N) | 1977–81 Rate | (N) | % change in rates | *Teratomas* 1972–76 Rate | (N) | 1977–81 Rate | (N) | % change in rates | *Germinomas and teratomas* 1972–76 Rate | (N) | 1977–81 Rate | (N) | % change in rates |
|---|---|---|---|---|---|---|---|---|---|---|---|---|---|---|---|
| *Testis* | | | | | | | | *Rates/100,000* | | | | | | | |
| 0–14 | 0.00 | (0) | 0.00 | (0) | — | 0.18 | (9) | 0.12 | (6) | −33.3 | 0.18 | (9) | 0.12 | (6) | −33.3 |
| 15–24 | 0.80 | (24) | 1.19 | (36) | +48.8 | 3.54 | (106) | 4.05 | (122) | +14.4 | 4.34 | (130) | 5.24 | (158) | +20.7 |
| 25–34 | 3.20 | (85) | 4.48 | (121) | +40.0[a] | 2.97 | (79) | 5.30 | (143) | +78.4[b] | 6.16 | (164) | 9.78 | (264) | +58.8[a] |
| 35–44 | 3.42 | (79) | 3.16 | (74) | −7.6 | 1.38 | (32) | 1.54 | (36) | +11.6 | 4.80 | (111) | 4.70 | (110) | −2.1 |
| 45–54 | 1.85 | (40) | 1.69 | (36) | −8.6 | 0.88 | (19) | 0.52 | (11) | −40.9 | 2.72 | (59) | 2.20 | (47) | −19.1 |
| 55–64 | 1.04 | (16) | 1.60 | (24) | +53.8 | 0.26 | (4) | 0.00 | (0) | — | 1.30 | (20) | 1.60 | (24) | +23.1 |
| 65–74 | 0.74 | (6) | 1.15 | (9) | +55.4 | 0.37 | (3) | 0.00 | (0) | — | 1.11 | (9) | 1.15 | (9) | +3.6 |
| 75+ | 1.14 | (5) | 0.71 | (3) | −37.7 | 0.23 | (1) | 0.48 | (2) | +108.7 | 1.38 | (6) | 1.19 | (5) | −13.8 |
| All ages | 1.42 | (255) | 1.68 | (303) | +18.3 | 1.41 | (253) | 1.77 | (320) | +25.5[a] | 2.83 | (508) | 3.44 | (623) | +21.6[a] |
| *Ovary* | | | | | | | | Rates/100,000,000 | | | | | | | |
| 0–14 | 0.41 | (2) | 0.40 | (2) | −2.4 | 2.04 | (10) | 1.98 | (10) | −2.9 | 2.44 | (12) | 2.38 | (12) | −2.4 |
| 15–24 | 2.23 | (7) | 5.04 | (16) | +126.0 | 2.23 | (7) | 3.15 | (10) | +41.2 | 4.45 | (14) | 8.18 | (26) | +83.8 |
| 25–34 | 0.40 | (1) | 4.32 | (11) | +980.0[a] | 0.40 | (1) | 3.14 | (8) | +685.0[a] | 0.80 | (2) | 7.47 | (19) | +833.8[b] |
| 35–44 | 0.46 | (1) | 0.00 | (0) | — | 0.92 | (2) | 0.91 | (2) | −1.1 | 1.38 | (3) | 0.91 | (2) | −34.0 |
| 45–54 | 0.90 | (2) | 0.92 | (2) | +2.2 | 1.80 | (4) | 1.38 | (3) | −23.3 | 2.70 | (6) | 2.29 | (5) | −15.2 |
| 55–64 | 0.00 | (0) | 1.24 | (2) | — | 1.76 | (3) | 1.86 | (3) | +5.7 | 1.76 | (3) | 3.10 | (5) | +76.1 |
| 65–74 | 0.00 | (0) | 0.88 | (1) | — | 0.85 | (1) | 0.88 | (1) | +3.5 | 0.85 | (1) | 1.76 | (2) | +107.0 |
| 75+ | 0.00 | (0) | 0.00 | (0) | — | 0.00 | (0) | 1.25 | (1) | — | 0.00 | (0) | 1.25 | (1) | — |
| All ages | 0.70 | (13) | 1.82 | (34) | +160.0[a] | 1.50 | (28) | 2.03 | (38) | +35.3 | 2.20 | (41) | 3.84 | (72) | +74.5[a] |

[a] $P<0.05$ (using Mantel-Haenszel Chi Square test corrected for continuity).
[b] $P<0.001$.

Other evidence suggesting an increasing incidence in germ cell tumours was reported by Birch *et al.* (1982) using data from the Manchester Children's Tumour Registry from 1954–78. According to their findings, there was a significant increase in the incidence of all malignant germ cell tumours from 1 to almost 4 per million person years. This finding, however, is not strictly comparable to the data in this report since it is restricted to children under 15 of both sexes and includes tumours in extragonadal sites as well as the gonadal tumours. Nevertheless, it is an important confirmation of an increasing secular trend in these tumours.

The increasing secular trends for both ovarian and testicular germ cell tumours noted in this report suggest that they may have aetiologic factors in common. We therefore compared other descriptive data on gonadal germ cell tumour occurrence in men and women in Los Angeles County. A comparison of the average annual age-specific incidence rates by histologic type derived from the entire ten year period (1972–81) showed that, in general, the rates for teratomas have an earlier age peak by about 10 years than do the rates for germinomas for both males and females (Figure 1). Specifically, for males, the rates for seminomas of the testis peak in the 30–34 age group while the highest rate for the teratomas occurs at 20–24 years of age. For females, a similar phenomenon is shown, although both peaks occur about 10 years earlier than for males, with the highest rate for teratomas in the 10–14 year age group followed by the peak rate for the dysgerminomas in the 20–24 age group. For males, we have proposed that this observation would be consistent with a common cellular origin of all testicular germ cell tumours if the rate of cell division, or perhaps other age-related host factors, was an important determinant of histologic pattern (Henderson *et al.*, 1983).

The earliest tumours in females occur at about age 8 which corresponds closely to the onset of an increase in circulating gonadotropins which occurs early in puberty (Swerdloff, 1978). The fact that the tumours in females occur earlier than in males is consistent with an earlier mean puberty in females by about two years (Marshall and Tanner, 1970), although this may not be a complete explanation. Another possible reason for the earlier age peak in females may be the higher levels of FSH and LH during the first two years of life in females, as compared to males, and the more rapid growth of the prepubertal ovary *vs* the prepubertal testis (Fairman and Winter, 1971).

Other than the large peaks in incidence in adolescence and young adulthood, females show an increase on incidence rates beginning around age 40 (a phenomenon also shown in data from the U.S. Third National Cancer Survey (Weiss *et al.*, 1977)), while males do not. In contrast, males show a very small early peak in the under 5 age group for the teratomas, a finding which has also been noted by others (Li and Fraumeni, 1972).

The high risk in whites, as compared to blacks, for testis cancer has been well documented both in

**Figure 1** Age-specific incidence rates of malignant gonadal germ cell tumours by sex and histologic type: Los Angeles County 1972–1981. (a) males (testis); (b) Females (ovary). (●) Germinomas; (○) Teratomas.

Los Angeles and elsewhere (Ross *et al.*, 1979; Schottenfeld *et al.*, 1980; Davies, 1981). Over the 10 year period 1972–81, the average annual age-adjusted incidence rate for testis cancer in non-Hispanic whites in Los Angeles was 3.5/100,000 as compared to 0.5/100,000 in blacks. Although whites also had a higher rate of malignant ovarian germ cell tumours in Los Angeles (0.33/100,000 in whites *vs* 0.19/100,000 in blacks), the magnitude of the difference is considerably less than for testicular tumours. In fact, Weiss *et al.* (1977) reporting data from the U.S. Third National Cancer Survey actually observed a higher rate in blacks (0.40/100,000) than whites (0.29/100,000).

High socioeconomic status is also a well established risk factor for testis cancer (Ross *et al.*, 1979; Schottenfeld *et al.*, 1980; Davies, 1981). Because of the small number of cases of malignant ovarian tumours, the association of this tumour with social class is difficult to study for females. Available data from the CSP suggest that malignant ovarian germ cell tumours may also have a positive relationship with social class, but that the strength of the association is considerably weaker than for testicular tumours.

Although some differences exist between the descriptive epidemiologies of germ cell tumours of the ovary and testis, the common increasing secular trends in younger age groups and the similarities between the tumours in age-specific incidence patterns suggest that they may share at least some aetiologic factors. For testis cancer, we have proposed that the initial event leading to germ cell tumours occurs *in utero* (Depue, *et al.*, 1983). Endogenous or exogenous events, probably hormonal in nature, lead to a permanent alteration in the primordial germ cells. These remain dormant until stimulated to multiply by rising levels of FSH and LH. The data presented for ovarian germ cell tumours may suggest a similar phenomenon in females and provides one possible avenue for further research.

This work was performed under Grant CA1 7054 from the National Cancer Institute, National Institutes of Health.

### References

BIRCH, J.M., MARSDEN, H.B. & SWINDELL, R. (1982). Pre-natal factors in the origin of germ cell tumours of childhood. *Carcinogenesis,* **3,** 75.

CLEMMESON, J. (1968). A doubling of morbity from testis carcinoma in Copenhagen, 1943–62. *Acta Pathol. Microbial. Scand.,* **72,** 348.

DAVIES, J.M. (1981). Testicular cancer in England and Wales: Some epidemiological aspects. *Lancet,* **i,** 929.

DEPUE, R.H., PIKE, M.C. & HENDERSON, B.E. (1983). Estrogen exposure during gestation and risk of testicular cancer. *J. Natl Cancer Inst.,* **71,** (in press).

FAIMAN, C. & WINTER, J.S.D. (1971). Sex differences in gonadotrophin concentrations in infancy. *Nature,* **232,** 130.

HENDERSON, B.E., GORDON, R.J., MENCK, H., SOOHOO, J., MARTIN, S.P. & PIKE, M.C. (1975). Lung cancer and air pollution in south central Los Angeles County. *Am. J. Epidemiol.,* **101,** 477.

HENDERSON, B.E., ROSS, R.K., PIKE, M.C. & DEPUE, R.H. (1983). Epidemiology of testis cancer. In: *Urological Cancer,* (Ed. Skinner), New York: Grune and Stratton, p. 237.

HISSERICH, J.C., MARTIN, S.P. & HENDERSON, B.E. (1975). An areawide reporting network. *Public Health Rep.,* **90,** 15.

LI, F.P. & FRAUMENI, J.F. Jr. (1972). Testicular cancer in children: epidemiologic characteristics. *J. Natl Cancer Inst.,* **48,** 1575.

MACK, T.M. (1977). Cancer surveillance program in Los Angeles County. *Natl Cancer Inst. Mono.,* **47,** 99.

MARSHALL, W.A. & TANNER, J.M. (1970). Variations in the pattern of pubertal changes in boys. *Arch. Dis. Childhood,* **45,** 13.

NOVAK, E.R. & WOODRUFF, J.D. (1979). *Novak's Gynecologic and Obstetric Pathology,* Eighth Edition. Chap. 25. Philadelphia: Saunders.

ROSS, R.K., McCURTIS, J.W., HENDERSON, B.E., MENCK, H.R., MACK, T.M. & MARTIN, S.P. (1979). Descriptive epidemiology of testicular and prostatic cancer in Los Angeles. *Br. J. Cancer,* **39,** 284.

SCHOTTENFELD, D., WARSHAUER, M.E., SHERLOCK, S., ZAUBER, A.G., LEDER, M. & PAYNE, R. (1980). The epidemiology of testicular cancer in young adults. *Am. J. Epidemiol.,* **112,** 232.

SWERDLOFF, R.S. (1978). Physiological control of puberty. *Med. Clin. North Am.,* **62,** 351.

TEILUM, G. (1976). *Special Tumours of Ovary and Testis and Related Extragonadal Lesions.* 2nd ed., p. 377, Philadelphia: Lippincott.

WEISS, N.S., HOMOCHUK, T. & YOUNG, J.C. Jr. (1977). Incidence of the histologic types of ovarian cancer; the U.S. Third National Cancer Survey 1969–71. *Gyn. Oncol.,* **5,** 161.


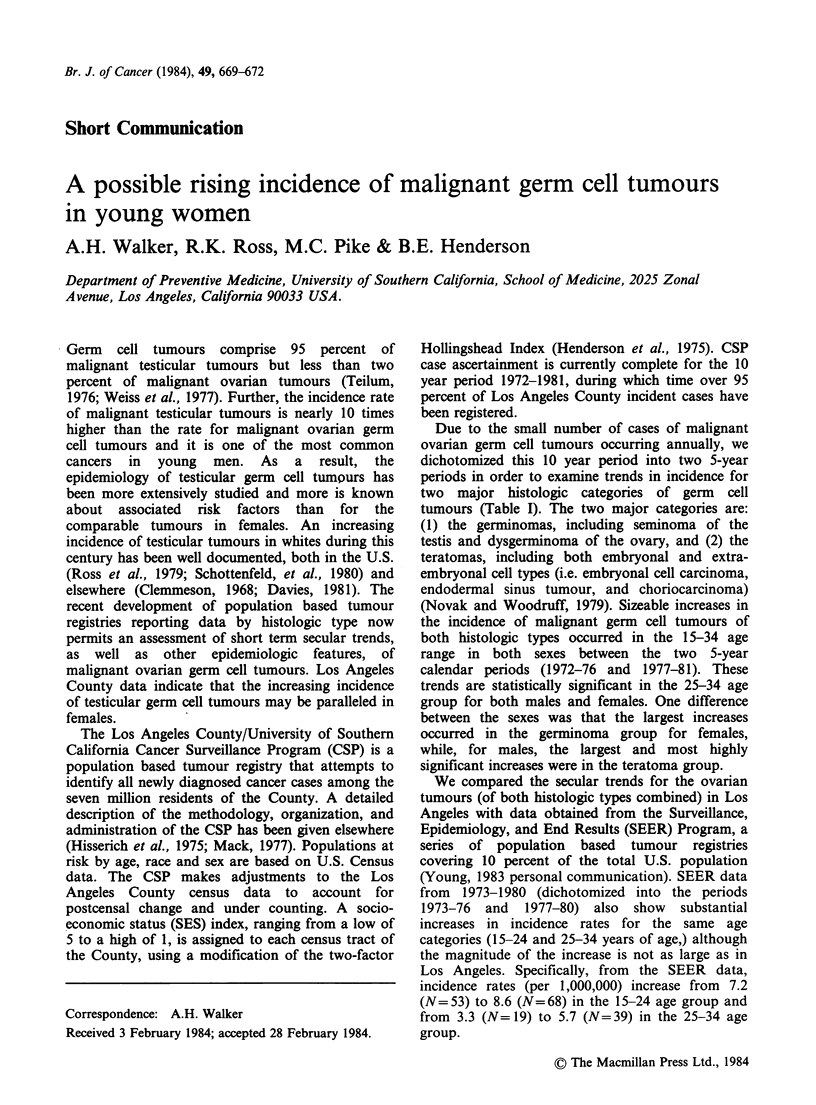

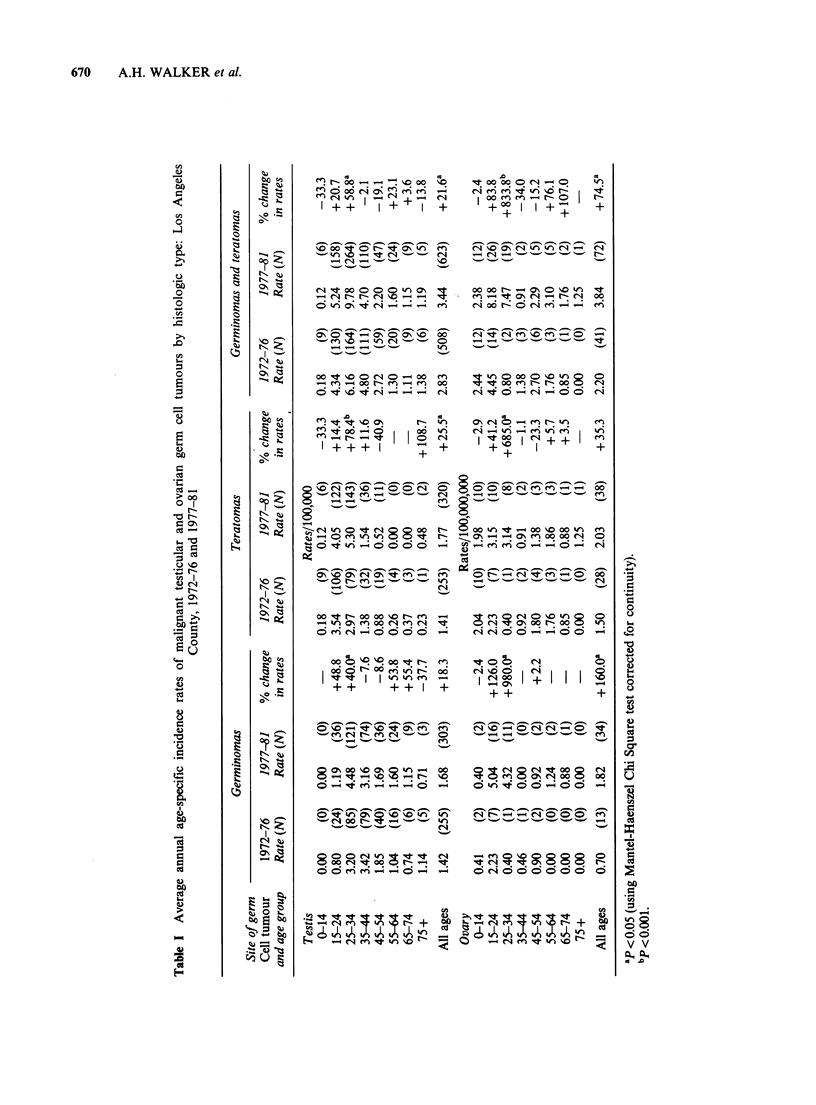

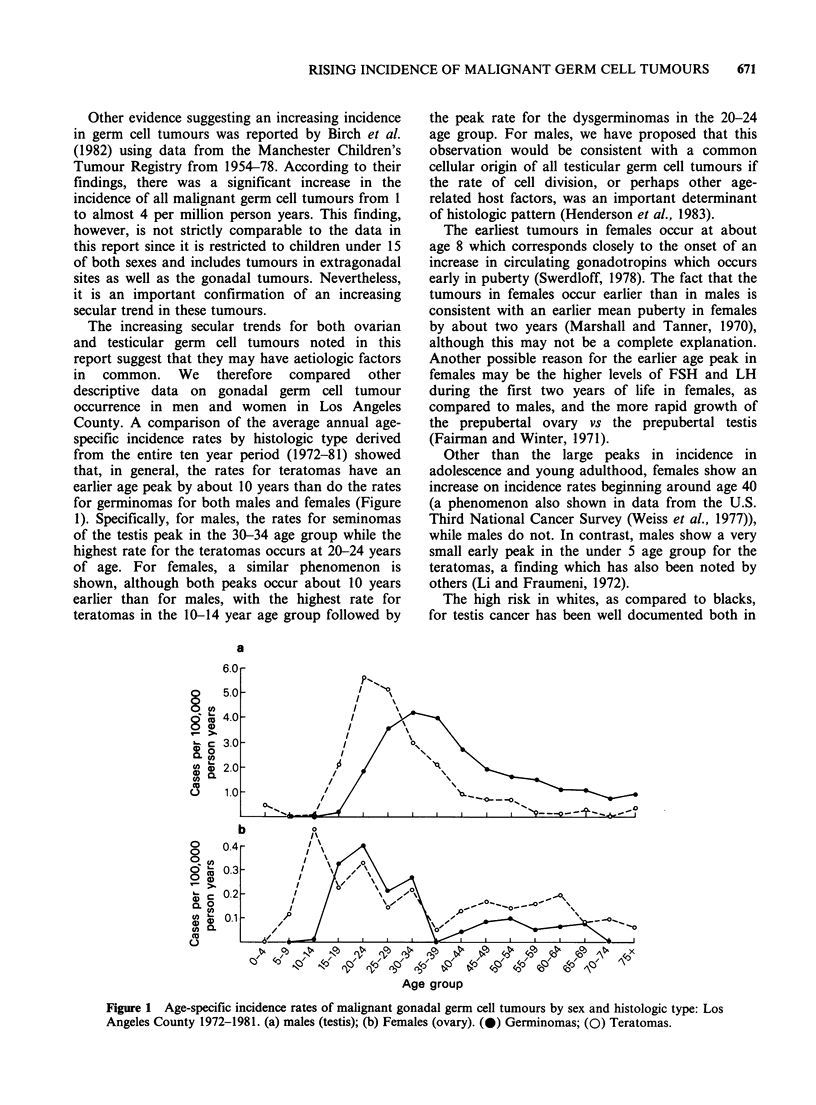

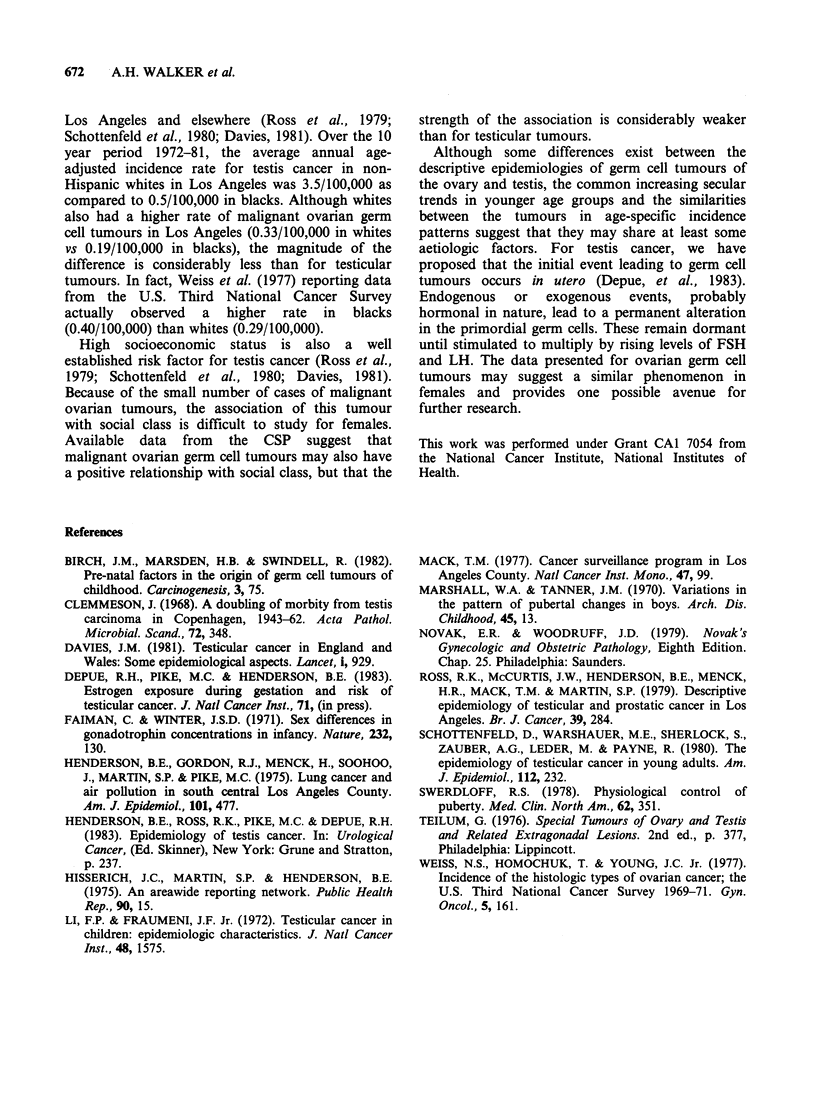


## References

[OCR_00482] Birch J. M., Marsden H. B., Swindell R. (1982). Pre-natal factors in the origin of germ cell tumours of childhood.. Carcinogenesis.

[OCR_00492] Cumming A. M., Davies D. L. (1979). Intravenous labetalol in hypertensive emergency.. Lancet.

[OCR_00501] Faiman C., Winter J. S. (1971). Sex differences in gonadotrophin concentrations in infancy.. Nature.

[OCR_00506] Henderson B. E., Gordon R. J., Menck H., Soohoo J., Martin S. P., Pike M. C. (1975). Lung cancer and air pollution in southcentral Los Angeles County.. Am J Epidemiol.

[OCR_00518] Hisserich J. C., Martin S. P., Henderson B. E. (1975). An areawide cancer reporting network.. Public Health Rep.

[OCR_00523] Li F. P., Fraumeni J. F. (1972). Testicular cancers in children: epidemiologic characteristics.. J Natl Cancer Inst.

[OCR_00528] Mack T. M. (1977). Cancer surveillance program in Los Angeles County.. Natl Cancer Inst Monogr.

[OCR_00532] Marshall W. A., Tanner J. M. (1970). Variations in the pattern of pubertal changes in boys.. Arch Dis Child.

[OCR_00542] Ross R. K., McCurtis J. W., Henderson B. E., Menck H. R., Mack T. M., Martin S. P. (1979). Descriptive epidemiology of testicular and prostatic cancer in Los Angeles.. Br J Cancer.

[OCR_00548] Schottenfeld D., Warshauer M. E., Sherlock S., Zauber A. G., Leder M., Payne R. (1980). The epidemiology of testicular cancer in young adults.. Am J Epidemiol.

[OCR_00554] Swerdloff R. S. (1978). Physiological control of puberty.. Med Clin North Am.

[OCR_00563] Weiss N. S., Homonchuk T., Young J. L. (1977). Incidence of the histologic types of ovarian cancer: the U.S. Third National Cancer Survey, 1969-1971.. Gynecol Oncol.

